# Description of a Collaborative Rural Dental Scholar Program

**DOI:** 10.1002/jdd.13859

**Published:** 2025-03-06

**Authors:** Carly Timmons McKenzie, Paul Drake Lavender

**Affiliations:** ^1^ Department of Clinical and Community Sciences Behavioral and Population Sciences Division, School of Dentistry University of Alabama at Birmingham Birmingham Alabama USA; ^2^ Department of Family, Internal, and Rural Medicine College of Community Health Sciences University of Alabama Tuscaloosa Alabama USA

**Keywords:** dental workforce, health care access, oral health, oral health care for the underserved, pathway program, professional interest, professional student, rural

## Abstract

**Purpose:**

Two educational institutions, The University of Alabama at Birmingham School of Dentistry and The University of Alabama College of Community Health Sciences, partnered to collaborate with representatives from a state agency and organized dentistry to, with legislative support, create and implement a coordinated series of initiatives designed to increase Alabama's rural dental workforce.

**Methods:**

This interrelated, long‐term program has three primary components: (1) Rural Dental Health Scholar residential summer program for rising high school seniors; (2) Rural Dental Scholar (RDS) pathway program for dental students featuring a pre‐matriculation master's curriculum; and (3) financial support by Alabama's Board of Dental Scholarships (BDS) for participation in said programs and service awards for practice in an eligible rural area following dental school.

**Results:**

A total of 31 students have participated across all aspects of this program, and a combined total of $3.39 M directly supports program participants. Nine RDSs are pathway participants across two academic years. The inaugural Rural Dental Health Scholar program in Summer 2024 included six rising high school seniors. The BDS has successfully contracted 16 future rural dentists.

**Conclusions:**

This multifaceted partnership across various educational, professional, and governmental entities is a much‐needed intervention intended to address the worsening rural dentist crisis in Alabama. Long‐term outcomes tracking will inform degree of success of these new initiatives in recruiting and retaining dentists to rural communities in need.

## 1 Introduction

The dentist shortage in America's rural areas is the primary workforce challenge in oral healthcare [1]. Although figures vary, the estimated 11% of dentists serving rural areas nationally fall short of the proportion of Americans residing in such communities [2, 3]. Approximately 11% of the state's dental schools’ graduates practice in rural areas [4], but almost half of Alabama's residents reside in rural communities [5]. Although the geographic distribution of Alabama's dentists aligns with national data, the state's high degree of rurality coupled with an aging rural dental workforce suggests that the inadequate dentist‐to‐patient ratio in rural areas will worsen [3, 5, 6].

The recruitment and retention of rural dental providers are a complex issue, but several factors are predictive of service in small towns. First, dentists with rural upbringing are more likely to settle in similar rural areas [1, 7, 8]. Second, dental students with a high degree of familiarity and comfort with rural practice and life, via either clinical experiences or mentorship, are more likely to practice in small towns [8, 9, 10, 11]. Therefore, the common factor among future rural dental providers appears to be knowledge of rural life, often developed via personal experiences and supplemented by mentorship.

As educators, researchers, policymakers, and organized dentistry representatives grapple with the challenge of enhancing Alabama's rural dental workforce, much remains unknown. Although previous research indicates that students aim to treat low‐income rural populations [12], practical considerations may thwart intentions. For example, many rural communities have lost population in recent years [13]; so how do perceptions of practice viability and thriving communities influence location decisions? Among early‐career dentists, to what degree are the frequently cited concerns of educational and employment opportunities within rural communities [14] viewed as problematic? Furthermore, how much can the lower cost of living in many rural communities offset perceived financial challenges such as student loan debt, needed investment capital, and high rates of uninsured, low‐income rural Alabamians [3, 15]?

These issues are complex, important, and beyond the purview of most educational institutions’ missions and resources. However, professional schools may deploy interventions intended to alleviate the problem of a shrinking rural healthcare workforce. With organized dentistry and state legislative support, the following three separate state entities partnered to implement a multifaceted intervention: the dental school, a sister higher education institution, and a state agency. The aim of this study is to investigate initial outcomes of this program's implementation.

## 2 Materials and Methods

This program incorporated three interrelated components: Rural Dental Health Scholars residential summer program for rural high school students interested in dentistry, Rural Dental Scholars (RDSs) pathway program featuring a Rural Community Health Master's degree prior to dental school matriculation, and Board of Dental Scholarships (BDS) awards that provide financial support for program participants and incentivize rural service commitment after graduation. The appropriate Institutional Review Board (IRB) deemed this study exempt (IRB‐300013803).

### 2.1 Participants

Three separate entities participated in all three of the programs that composed this effort are The University of Alabama at Birmingham School of Dentistry (UAB SOD), The University of Alabama College of Community Health Sciences (UA CCHS), and the Alabama (BDS state agency. The Alabama cities of Birmingham, Tuscaloosa, and Montgomery housed these entities, respectively. In addition, the Alabama Dental Association supported these efforts in tandem with state government to ensure budget allocations for programmatic and administrative support.

### 2.2 Program Design

#### 2.2.1 Rural Dental Health Scholars High School Program

The Rural Dental Health Scholar residential program targeted rising high school seniors who expressed interest in dentistry and resided in a rural Alabama community. These scholars participated with other rural high schoolers interested in healthcare careers during a 6‐week residential summer program hosted at UA in Tuscaloosa. Applicants to the program submitted an online application, high school transcript, and letters of recommendation. Along with demographic and academic information, questions prompted candidates to detail their interest in, and motivation for, exposure to healthcare.

Modeled after a long‐standing, successful program designed to promote rural primary care medicine, scholars attended UA during the first summer term and enrolled in two classes: General Chemistry and Creative Writing. Students were housed in campus dorms, and all expenses were covered during their experience, enabling participation regardless of financial status. The Alabama BDS financially supported scholars’ participation.

#### 2.2.2 Rural Dental Scholars Pathway Program

This 5‐year pathway program sought to increase the number of future dental providers in underserved areas by helping qualified rural dental school applicants strengthen their academic foundation in the biomedical sciences, further develop study skills, and acquire knowledge related to rural health. This program modeled the Rural Medical Scholars Program framework, a successful initiative developed by the UA CCHS in partnership with the UAB Heersink School of Medicine (HSOM) [16]. Fifty‐eight percent of the graduates of the Rural Medical Scholars Program have gone into rural primary care practice over its 29‐year history. A 1‐year master's program, executed by the UA CCHS in Tuscaloosa, preceded dental school matriculation and focused on Rural Community Health. Although RDSs funded their own master's program year, the Alabama BDS awarded a 1‐year $20,000 scholarship for each matriculated RDS during the first year of dental school to help offset resource investment. Although this scholarship does not cover the entire cost of the pre‐matriculation master's year, it does represent about 58% of the total cost of attendance.

#### 2.2.3 Board of Dental Scholarships Service Awards

A financial service award for commitment to future practice in a rural Alabama community constitutes the third and final component of these interrelated initiatives. The Alabama BDS refocused efforts and consolidated resources in recent years to provide attractive financial incentives promoting service in areas of need. All current dental students are eligible, although students further along in their education often have more well‐developed practice intentions. Awardees agree to practice in an approved location for a minimum time (typically 5 years) but choose specifically where to practice among a list of provided eligible locations. Multiple workforce factors determine location eligibility, including dentist‐to‐population ratio, percent of dentists above 65 years of age, and dentist shortage index.

### 2.3 Outcomes

This study focused on two primary outcomes: number of participants in each phase of the program and the amount of financial investment allocated to said participants.

## 3 Results

As of this writing, a total of 31 students engaged in this multifaceted program. A combined total of $3.39 M has been awarded to directly support program participants.

### 3.1 Rural Dental Health Scholars High School Program

#### 3.1.1 Participants

Representatives from UAB SOD and UA CCHS jointly participated in recruitment and selection of participants. Rural Dental Health Scholar evaluation criteria included eligibility, defined as rural residence or attendance at a rural high school, the candidate's voluntary expression of interest in dentistry, and potential to benefit from program participation. Six of seven accepted candidates participated during Summer 2024 to compose the initial cohort of Rural Dental Health Scholars.

#### 3.1.2 Financial Investment

The Alabama BDS invested $15,000 in each Rural Dental Health Scholar to cover tuition, room and board, and associated program expenses. For the initial cohort, the BDS allocated a total of $90,000 for all six participants.

### 3.2 Rural Dental Scholars Program

#### 3.2.1 Participants

Representatives from UAB SOD and UA CCHS jointly participated in recruitment and selection of participants. UAB SOD DMD admissions committee members identified potential RDS candidates during the regular DMD admissions cycle. These candidates were then separately evaluated and interviewed by CCHS representatives. Applicants had the option to self‐report program interest during the DMD admissions cycle. Eligibility criteria included Alabama residence, extended lived experience in a rural community, and voluntary, seemingly genuine expression of interest in eventual rural practice in the state. Interest in rural practice is typically evaluated at multiple points throughout the portfolio via the written personal statement, secondary application, dental experience in rural practice settings, letters of recommendation, and interview.

A total of nine RDSs accepted offers for this program. The conditional DMD offer allowed matriculation as a D1 upon successful completion of the stated requirements associated with the Rural Community Health Master's program. These included a minimum cumulative 3.50 grade point average during the master's year. The first cohort of four RDSs all completed the master's program in May 2024 and matriculated as D1's in July 2024. Five RDS candidates enrolled in the Rural Community Health Master's curriculum for the 2024−2025 academic year with expected dental school matriculation in July 2025.

#### 3.2.2 Financial Investment

The Alabama BDS invested $20,000 in each RDS via a scholarship awarded during the D1 year. This award helped offset each individual's additional expenses required by master's program participation. In addition, as UA CCHS expanded the existing Rural Medical Scholars program to accommodate RDSs, the Alabama Dental Association successfully collaborated with state lawmakers to allocate funds to help support the costs of administering this program.

### 3.3 Board of Dental Scholarships Service Awards

#### 3.3.1 Participants

Beginning in the 2020−2021 academic year, the BDS partnered with UAB SOD to advertise award availability, recruit applicants, and select candidates. Eligibility criteria included current enrollment at any dental school, good academic standing, and Alabama residency as defined by the state (not limited to in‐state residency as defined by the UA system). Candidates submitted an online application to UAB SOD representatives within a defined window and eligible applicants interviewed with BDS members during their regularly scheduled, biannual meetings. A total of 16 service awardees have been successfully contracted. As of this writing, four of these service awardees are currently practicing in identified areas of need, eight awardees are graduating dental school in 2025 with plans to enter rural practice, and four students have been offered, but funds have not yet been issued. Currently, approximately 23 Alabama counties qualify as eligible locations for the BDS award.

#### 3.3.2 Financial Investment

Selected candidates received a lump sum of $180,000, payable directly to the student, and are contractually obligated to serve at least 5 years of service in an Alabama small town/rural community in need of a dentist. This award increased to $200,000 in 2024, and awardees still in school retroactively received the increased amount. The award money is in addition to any regular earnings and does not replace or constitute practice income. Awardees can use the funds; however, they wish but are encouraged to invest in practice facilities and/or repay educational loans. If a graduate does not meet the agreed‐upon service requirement in an approved rural location, the contract terms stipulate monetary repayment with interest and penalties.

Since the refocus during 2020−2021 on sizeable service awards to incentivize rural dental practice in Alabama, the BDS has awarded a total of $3.12 million as of this writing. Currently, the awardees’ actual or intended practice locations span 14 counties of need across the state, an area that includes over 506,000 residents [17]. Within eight of these dental shortage counties, the number of dentists has further decreased since 2017 [6]. Of these 14 counties, 10 counties house an aging dental workforce, defined as follows: one county reports 100% of dentists as over 60 years of age, 50%−59% of dentists in one county are 60+ years of age, four counties report no dentists less than 40 years of age, and four counties contain a single dentist younger than 40 years [6]. This illustrates that although the addition of any dentists at all in these underserved counties is much needed, enhancing the workforce with early‐career dentists is especially desirable.

## 4 Discussion

This three‐tiered approach creates a formalized pathway to rural dental practice in Alabama. Thus far, 31 students from Alabama's high schools, colleges, and dental schools are engaged in this program. Participation facilitates academic, financial, social, and administrative support and development among those interested in pursuing dentistry in an Alabama small town or rural community. The collaboration of organized dentistry, state government, and two educational institutions allows for resources to be efficiently and effectively targeted toward students in multiple phases of their professional journey.

Several issues are critical for the successful structure and implementation of these programs. First are candidate evaluation and selection benefits with input across entities. For the Rural Dental Health Scholar and Rural Dental Scholar pathway programs, representatives from both UAB SOD and UA CCHS participate in recruitment and selection. Eligibility criteria include rural residency in Alabama (high school attendance or at least 8 years in a rural community), explicit interest in practicing dentistry in a rural/small town community, and potential benefit from program participation. Determination of rurality is informed by formal federal definitions as well as knowledge of the local area's culture.

Second, successful academic development requires a holistic approach characterized by flexibility and individual support. For example, the RDS master's degree incorporates public health topics in addition to challenging science content as preparation for both rural practice and the academic rigors of professional school. The UA CCHS Rural Community Health Master's curriculum includes classes on health policy, rural environmental and occupational health, and graduate‐level biology courses including immunology, histology, physiology, and pharmacology. Program personnel selected this graduate science content after surveying the students regarding courses that most helped them successfully complete professional school coursework. In addition, the master's curriculum can be tailored to meet the specific academic needs of individuals.  For both programs, academic support systems are necessary. Performance is carefully monitored, and scholars have access to tutors to help ensure success in challenging classes.

Third, candidate development must be multifaceted and include development in other areas necessary for eventual success in professional school. All graduate students take a class focused on clinical correlations of the basic science courses as well as study skills, test‐taking strategies, and wellness plans. This mandatory course is co‐taught by clinicians and a doctorate‐level educational psychologist and helps students holistically prepare for the demands of professional school. In addition, rural healthcare interest is fostered with targeted activities. Rural Dental Scholar Master's students travel to rural communities and dental offices to experience the settings and personally interact with local dentists. Students also take a survey course, which reinforces their connection with rural communities through shadowing, a community health assessment of their hometown, and a capstone project impacting one identified issue. During the high school residential program, students participate in workshops, lectures, and lunch‐and‐learn sessions exposing them to dentistry and other healthcare careers.

Prior experiences with the existing Rural Medical Scholars Program afford valuable insights. For example, academic preparation and continued rural community connection surely play important roles in the successful completion of professional school and eventual rural practice. However, Rural Medical Scholar graduates consistently cite the importance of the pre‐matriculation year's cohort as the program's primary advantage. Students form tight bonds during the master's year with colleagues who have similar lived experiences and career goals. These peer connections appear to mitigate the influences of urban living and/or specialization interests that may seduce students in urban professional school environments. In addition, this foundational cohort's importance may persist as support of and connection to colleagues encountering similar experiences may help increase long‐term retention rates among rural dental practitioners [14].

In addition to the pre‐matriculation curriculum and cohort support, activities in the dental school curriculum support the maintenance of scholars’ connections to rural communities. First, RDS relate their research and service components of the SOD 4‐year portfolio [18] to an issue, organization, or population relevant to Alabama's rural/small town communities. Other students interested in exploring potential practice in a rural area/small town in Alabama, especially candidates interested in the BDS service awards, are also encouraged to conduct research and/or service activities relevant to such communities. Second, RDS are afforded priority voluntary participation in the Area Health Education Center (AHEC) scholars program, an external interdisciplinary initiative focused on developing rural clinicians. Third, students participate in external clinical rotations both situated in rural areas and those serving patients from surrounding rural communities. Prior research pinpoints well‐supported, high‐quality clinical experiences in rural communities as important for rural practice intentions [8, 9, 19]. In these ways, the RDS thus continue to acquire targeted experiences with rural populations. Multiple mandatory external rotations occur in rural communities across the state, and the school also offers voluntary rotations in rural areas for eligible students. Finally, the SOD facilitates connections with rural practitioners via a mentor network. Dentists practicing in rural communities connect with interested SOD students, including RDS, to serve as mentors and information sources about living and working in Alabama's small towns. These relationships begin during the master's year as scholars are matched with shadow sites, some of which are located in rural communities. In addition, the SOD Alumni Office connects students with practitioner mentors during their time at the SOD, although these relationships are not formally managed through the school. This mentorship and accompanying exposure to knowledge about rural life appear to be a key factor in eventual rural practice [11].

Program participants continue to receive support and encouragement for rural practice after dental school. RDS service award applicants will be afforded priority consideration in future BDS financial allocation decisions as they are positively viewed by BDS members as evidence of consistent rural practice intentions. This financial incentive provided to students well‐suited for rural practice helps graduates establish roots in underserved communities, thus increasing the odds of retention as demonstrated elsewhere [19, 20].

There are several factors that may limit the effectiveness and success of these programs. First, the rural applicant pool is highly variable with yearly fluctuations in quantity and quality. The population trends in both rural communities and college enrollment may present challenges for the future. Second, candidates’ ability to be professionally successful is untested. The continued support likely needed in dental school, for the development of both academic endeavors and rural connections, may be inadequate. Third, funding is dependent on state government priorities, availability, and personnel. As program continuation depends on this financial support, such uncertain factors threaten sustainability.

As these programs are in their infancy, future research should explore outcomes so that efforts can be appropriately tailored. This program's success is defined by multiple outcomes of interest in addition to program participation and financial support. First, Rural Dental Health Scholars’ eventual application and successful admission to dental school are key success measures for the high school residential program. Next, RDSs’ successful master's completion and eventual matriculation at the SOD are of interest, in addition to their academic performance during dental school. Finally, the long‐term retention rate of BDS recipients in Alabama's rural communities is unknown. The following data points are especially important for tracking this key outcome, particularly among BDS awardees: a snapshot of how many providers are practicing in the state's rural counties, with a focus on early‐career dentists; a number of underserved counties with a dental provider added; program participants’ length of service in underserved rural areas, especially after defined contracted terms are fulfilled; and dentist‐to‐population ratios. Usage of dental services in said communities and oral health indicators are also eventual outcomes of interest, although measurement of workforce trends takes precedence for this particular program.

## 5 Conclusion

This collaborative effort of two universities, a state funding agency, organized dentistry representatives, and state legislators seeks to address the worsening issue of dentist shortages in Alabama's rural communities. This long‐term program incorporates three initiatives: (1) engagement of high school students from rural areas via the RDS summer program; (2) development of and investment in promising dental students hailing from and intending to practice in Alabama's rural communities via the RDS pathway program; and (3) financial support by Alabama's BDS for participation in said programs and service awards for practice in an eligible rural area following dental school. Uncertain factors such as continued political will, resource support and availability, personnel changes, and applicant pool may impact the sustainability and success of these programs. Although successfully implemented, future research must assess the effectiveness of these initiatives in recruiting and retaining dentists to Alabama's small towns and rural communities most in need.

## Conflicts of Interest

The authors declare no conflicts of interest.
